# Serological survey of canine parvovirus 2 antibody titres in breeding kennels in northern Italy

**DOI:** 10.1186/s12917-019-2085-4

**Published:** 2019-09-18

**Authors:** Ada Rota, Andrea Dogliero, Elvira Muratore, Paola Pregel, Angela Del Carro, Loretta Masoero

**Affiliations:** 1 Department of Veterinary Sciences, University of Turin, Largo Paolo Braccini 2-5, 10095 Grugliasco, Italy; 2 Practitioner and ECAR resident, Rawdat Al FarasHoubara Breeding Center (Ministry of Municipality&Environment), Doha, State of Qatar; 3 Istituto Zooprofilattico Sperimentale del Piemonte, Liguria e Valle d’Aosta, Via Bologna 148, 10154 Torino, Italy; 4 Practitioner, Torino, Italy

**Keywords:** Dog, Breeding kennel, Canine parvovirus, Antibody, Vaccination

## Abstract

**Background:**

Current guidelines recommend parvovirus revaccination of adult dogs no more frequently than every 3 years. The aim of this study was to determine the prevalence of dogs showing protective serum antibody titres against canine parvovirus 2 in breeding kennels in Northern Italy and to assess the effect of time from vaccination and the sex of the dog on antibody titres. The study was carried out on 370 animals of different breeds kept in 33 breeding kennels. Antibodies to canine parvovirus 2 in serum samples were measured with an indirect immunoenzymatic assay validated by the manufacturer in relation to the ‘gold standard’ haemagglutination inhibition test. The number of months that had elapsed since the last vaccination was calculated for each animal and categorized into the following classes: < 12 months; 13–24 months; 25–36 months; 37–48 months; and > 49 months.

**Results:**

The prevalence of ‘unprotected’ dogs was 4.6%. A satisfactory solid herd immunity was present in the majority of breeding kennels, although some vaccination failures were detected. A significant negative correlation was found between antibody titre and months since last vaccination. Comparable antibody titres were found in the first 3 years after vaccination. Although the antibody titre over time was not affected by the sex of the dog, ‘unprotected’ females had been vaccinated more recently than males with analogous low titres.

**Conclusions:**

Parvovirus revaccination of adult dogs every 3 years, as currently recommended, is also the appropriate recommendation for breeding kennels. Serological tests could be a useful tool to assess the effectiveness of vaccination.

## Background

Canine parvovirus type 2 (CPV-2) is the aetiological agent of a severe viral disease in dogs. It emerged as a dog pathogen in the late 1970s, when outbreaks of haemorrhagic gastroenteritis were observed in puppies and young dogs in kennels and shelters worldwide [1]. Moreover, the virus was demonstrated to be responsible for myocarditis in puppies [2]. CPV-2 is a nonenveloped single-stranded DNA virus that is closely related to feline parvovirus (FPV) but exhibits more rapid evolution [3]. Many antigenic variants (CPV-2a, CPV-2b and CPV-2c) have indeed completely replaced the original type-2 [3, 4]. Contagion occurs through the oronasal route, and the incubation period is three to 7 days [3]. CPV-2 can survive in the environment for more than a year, allowing the exposure of susceptible dogs to infected materials such as faeces, vomitus, or fomites. Virus shedding starts a few days prior to the occurrence of clinical signs, progressively declining 3–4 weeks postexposure [5].

Vaccination is the main method for controlling the disease, and modified live virus (MLV) vaccines are used to obtain long-term immunity. Maternally derived antibodies acquired via colostrum protect newborns during the first weeks of life and can interfere with vaccination [4]. Lifelong immunity to the diseases develops after natural CPV-2 infection/disease, while the persistence of antibodies in MLV-vaccinated dogs can last up to 10 years [6]. Current guidelinesrecommend parvovirus revaccination of adult dogs no more frequently than every 3 years [7] since the minimum duration of immunity after MLV vaccination may be even longer.

The aim of this study was to determine the prevalence of dogs with protective serum antibody titres against CPV-2 in medium-sized breeding kennels in Piedmont, Northern Italy and to investigate the effect of time from vaccination and the sex of the dog on antibody titres.

## Results

Four dogs, three females and a male, had never been vaccinated: three of them (age 11, 14 and 16 years), housed in the same kennel, had a titre lower than the cut-off titre (approximately 1:65), while the fourth dog (age 2 years), kept in a different kennel, had a titre of 1:184, which is deemed protective.

**Fig. 1:**
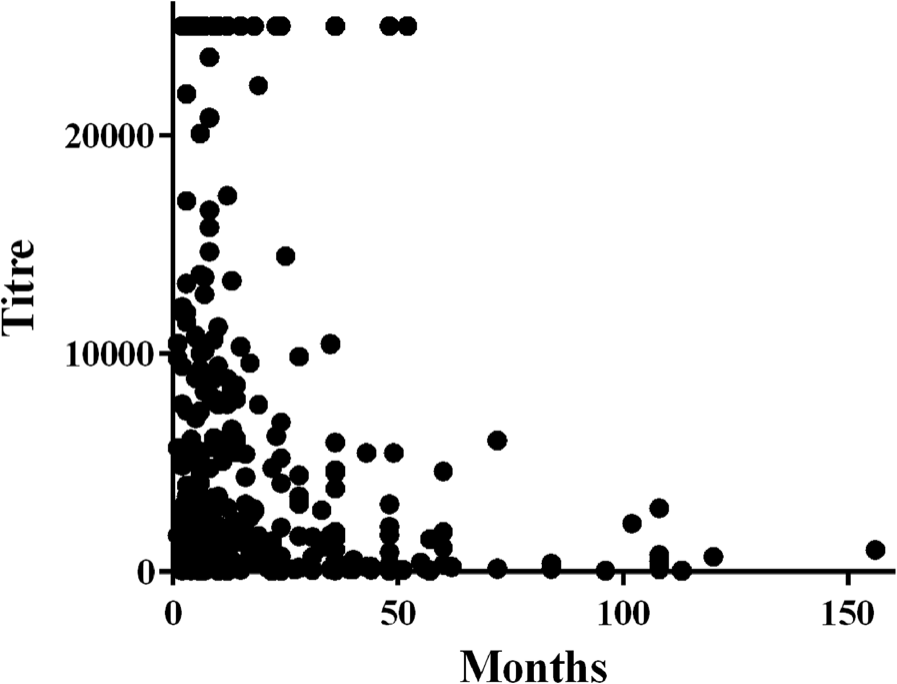
Correlation between antibody titre and the number of months since last vaccination (r = − 0.2048; *P* < 0.0001). An arbitrary value of 25,000 was attributed to out-of-scale antibody titres

**Fig. 2:**
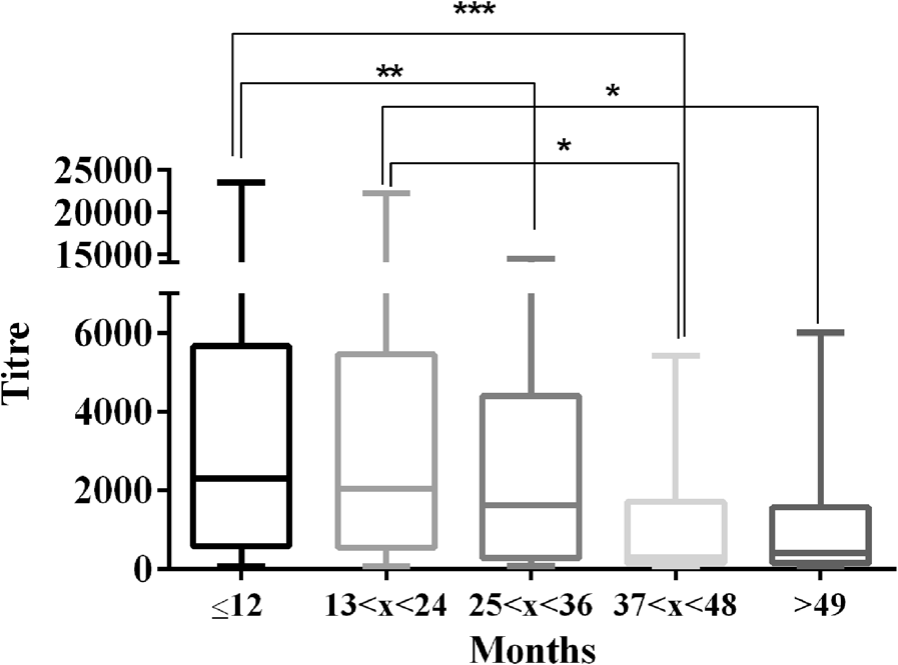
Comparison of antibody titres among different categories of time elapsed since last vaccination (Categories: < 12 months; 13–24 months; 25–36 months; 37–48 months; and > 49 months). (* = *P* < 0.05; ** = *P* < 0.01; and *** = *P* < 0.001)

In vaccinated animals, a significant negative correlation was found between antibody titre and the number of months since the last vaccination (Spearman r = − 0.2048; *P* < 0.0001) (Fig. 1). Time significantly affected the antibody titre (P < 0.0001), with comparable values in the first 3 years after vaccination (Fig. 2). Although the mean antibody titre was still protective even 49 months after the last vaccination, there was very large individual variation, and the median values dropped after the third year (Table 1); analogously, the percentage of unprotected animals was 2.7, 6.1 and 3.4 in the first 3 years and became 10.5 and 11.1 in the last two time categories, between 37 and 48 months and more than 49 months since the last vaccination, respectively.

**Table 1:** Serological titres of dogs in the five periods of time (months) since last vaccination (mean ± standard deviation and minimum, median, and maximum values) (N = number of animals for each time category)

Months	N	Antibody titre			
		Mean ± SD	Minimum	Median	Maximum
< 12	224	6009 ± 7589	61.4	2578	25,000
13 < x < 24	66	5071 ± 6906	65.7	2346	25,000
25 < x < 36	29	4485 ± 6653	80.9	1682	25,000
37 < x < 48	19	2279 ± 5667	61.8	334.1	25,000
> 49	27	2082 ± 4876	59.5	439.4	25,000

**Fig. 3:**
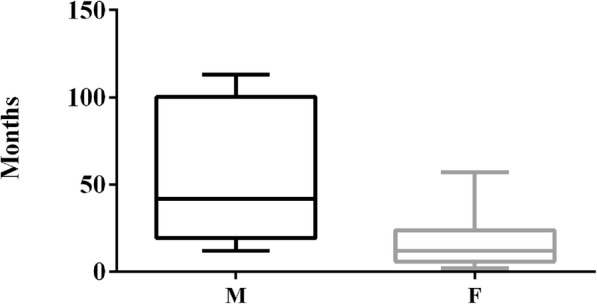
Comparison of the time elapsed since last vaccination between males and females showing lower than cut-off antibody titres (*P* = 0.0509)

Although the antibody titre over time was not affected by the sex of the dog, females with a titre lower than 1:100 tended to be vaccinated more recently than males with analogous low titres (*P* = 0.0509) (Fig. 3).

**Fig. 4:**
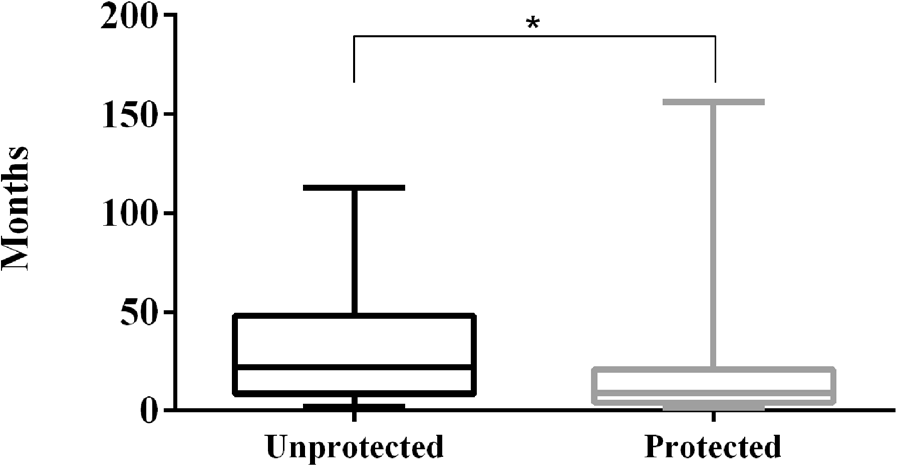
Comparison of the time elapsed since last vaccination between unprotected (lower than 1:100 titre) and protected animals. (* = *P* < 0.05)

In the 17 dogs in which the antibody titre was lower than 1:100, a longer period of time had elapsed since the last vaccination (*P* = 0.0126) in comparison to the “protected animals” (Fig. 4). The prevalence of ‘unprotected’ dogs was 4.6%, which was not significantly different between males (5.4%) and females (4.3%). Eight ‘unprotected’ dogs were older animals that had been vaccinated more than 3 years earlier; however, four young dogs, approximately 1 year of age, showed lower than the cut-off antibody titre, although they had been vaccinated less than 1 year earlier. A four-year-old German Shepherd, annually revaccinated, had an antibody titre lower than 1:100 2 months after the last vaccination. In the list of ‘unprotected’ dogs, two breeding kennels were represented with two and three dogs, respectively.

## Discussion

Canine parvovirus is highly feared in breeding kennels because the disease that it induces causes high morbidity and often mortality in puppies and young dogs. In addition to severe haemorrhagic gastroenteritis, puppies less than 3 months of age develop myocarditis, and CPV-2 myocardial infection is likely an underrecognized cause of cardiac damage in young dogs [8]. Collectively housed dogs can have a higher risk of parvovirus infection, depending on many variables such as population immunization, population density, sanitation of facilities, isolation protocols for new dogs, and turn-over rates. The virus is resistant to most disinfectants and is not easily removed from a contaminated environment, especially when organic material is present, or from soil and grass [9, 10].

Good hygienic practices in the kennels, including disinfection of all exposed surfaces and personnel, are primary control measures, given the ability of the virus to survive for a long time in the environment. Sodium hypochlorite represents an effective viricidal reagent, provided that contact time is at least 10 min [11].

CPV-2 antibody titres can be used to assess whether individual dogs are protected against infection: neutralizing antibody titres for CPV-2 are indeed recognized correlates of protection, so seropositive dogs above the cut-off value are deemed protected against infection [7].

A substantial proportion of dogs in the present study had protective antibody titres, so satisfactory solid herd immunity was detected in the majority of breeding kennels. Rather, unexpected findings are the presence of non-vaccinated adult dogs, especially when kept in breeding kennels: the vaccine against this infection is indeed part of the core vaccinations [12]. Current guidelines recommend parvovirus revaccination of adult dogs no more frequently than every 3 years, which is considered the minimum duration of immunity [7]. Our findings confirm that the antibody titre is similar in the first 3 years following vaccination, but in the following years, it still remains well above the cut-off in a large number of dogs, although the percentage of unprotected animals rises above 10% in the last two time categories (37 < x < 48; > 49).

The habit of yearly vaccination is still rather diffuse in the area of our investigation: for the majority of dogs, even when we excluded those younger than 1 year, less than 12 months had indeed elapsed since the last vaccination.

When dogs recover from natural infection/disease due to CPV-2, they develop lifelong immunity [6]. After initial MLV vaccination, the longest period of time that antibody was found to persist was 10 years for dogs kept in natural environments [6]. Additionally, in our dog population, there was one case of protective antibody titre 10 years after the last vaccination, and further, the case of a 14-year-old English Setter female that was still protected 13 years after the first and only vaccination. Notwithstanding single cases, at the population level, intervals of more than 4 years since the last vaccination were determined to be the main risk factors for the absence of CPV-2 antibodies [13].

Immunity following vaccination can vary among dogs, and our data confirm that older animals can show a decline in immunity, called “immunosenescence”, which may make them more susceptible to infectious diseases [6].

Due to the variable duration of immunity, current guidelines give the option to test the animals for seropositivity before blind revaccination [7]; rapid and simple serological test kits are available for in-practice use and can detect the presence of protective antibodies specific for canine distemper virus, canine adenovirus and CPV-2. The main limit to the regular use of the kits is their cost, which is equivalent to the cost of vaccination [12, 14].

Serological tests could be a useful tool to assess the effectiveness of vaccination in breeding kennels to check for unprotected animals and identify the reasons for vaccination failure. Among the dogs included in our study, some young animals did not show a protective serum antibody titre notwithstanding recent vaccination. For two dogs of different breeds and kept in the same breeding kennel, the more likely hypothesis is an improperly preserved/administered vaccine. Detection of this condition would be essential for a breeder to be able to correct inappropriate practices that could have dangerous effects. Dogs that fail to develop measurable antibody levels following adequate parvovirus vaccinations may be ‘genetic non-responders’ and represent another cause of vaccination failure: it is estimated that up to one in 1000 dogs may be genetic non-responders to CPV-2 [12]. The four-year-old German Shepherd that was revaccinated annually and had an antibody titre lower than the cut-off 2 months after the last vaccination is likely to be a genetic non-responder. The prevalence of one in 366 dogs, which is higher than what was reported [12], could be referred to as the genetic relatedness of the animals that represent the population of our investigation.

We did not record the type of the vaccine used for each dog, but we confirmed that it was a modified live virus vaccine from any of the major international manufacturers. In recent years, some concerns have arisen about the complete efficacy of CPV-2-based vaccines against the antigenic variants that have quickly and completely replaced the original type [3]. Vaccination of dogs with canine parvovirus type 2b would cross-protect against CPV-2a and CPV-2c, as well as against CPV-2 [15]. In the animals immunized with CPV-2, a substantial difference was found in the amount of serum-neutralizing activity towards the antigenic variants -2a, −2b, and -2c, which was lower than that towards the original type [1]. However, dogs that show a strong active immune response, demonstrated by very high antibody titres following repeated immunizations, are likely to be protected against the disease regardless of the variant [1].

## Conclusions

Our data show that the serological titre within 3 years since the last vaccination is generally far higher than the minimum protective titre. Serological tests could be used to monitor vaccination effectiveness in breeding kennels.

## Methods

### Animals

The study was carried out in 33 breeding kennels homogeneously distributed in the Piedmont region territory, North-West Italy, in 2018. The kennels were of small/medium size and housed a number of bitches of reproductive age, ranging from 3 to 15, that produced a number of litters ranging from 2 to 10 per year. The kennel history did not report episodes of parvovirus infection in the last 5 years. In a single kennel, a bitch and one of her puppies had died of parvovirus infection 3 years earlier. The dog population consisted of 370 animals, 257 females and 113 males. The mean age (±standard deviation) of the bitches and the dogs was (4.3 ± 2.9) and (4.8 ± 3.0), respectively, ranging from 8 months to 16 years for females and 11 months to 13 years for males.

All dogs were healthy and under veterinary control. The minimum age of inclusion in the study was 8 months, and 40–60% of the selectable animals were sampled in each kennel. The mean number of dogs tested in each breeding kennel was 7.8 (±6.5) females and 3.4 (±3.3) males, ranging from a minimum of 0 to a maximum of 15 males and from a minimum of 1 and a maximum of 25 females. Only breeds with an average weight higher than 8 kg were chosen to make blood collection easier and less stressful for the dogs. The represented breeds and the relative numbers are the following: Afghan hound (11), Airedale Terrier (5), AlpenlaendischeDachsbracke (7), Akita Inu (17), American Staffordshire terrier (7), AppenzellerSennenhund (13), Australian Shepherd (52), Bernese Mountain Dog (20), Bloodhound (3), Border Collie (7), Boxer (3), Clumber Spaniel (2), Czechoslovakian Wolfdog (3), Deutsch Kurzhaar (7), English Setter (33), French Bulldog (7), German Shepherd (36), Golden Retriever (36), HannoverscherSchweisshund (2), BraccoItaliano (2), Miniature American Shepherd (2), Pointer (3), Poodle (3), Riesenschnauzer (4), Romagna Water Dog (7), Rottweiler (15), Saarloos Wolfhound (4), Scotch Collie (6), SegugioMaremmano (1), Siberian Husky (2), ShibaInu (10), SpinoneItaliano (7), Staffordshire Bull terrier (14), Vizsla (5), Zwergpinscher (4), Weimaraner (3), White Swiss Shepherd Dog (5), and Whippet (2).

For each animal, sex, age and time of the last vaccination against parvovirus were recorded.

The study was carried out in accordance with the guidelines for the care and use of animals of the Department of Veterinary Sciences of the University of Turin (Italy) and with the consent of the dog owners.

### Sample collection

Blood samples (2 ml) were collected by cephalic venipuncture into 8 ml blood collection tubes (Vacuette®, Z Serum Sep Clot Activator, Greiner Bio-One North America Inc., North Carolina, USA) and carried to the laboratory at 4 °C within 5 h of collection.

Serum was separated by centrifugation (3500 rpm/min for 10 min) and stored frozen at − 20 °C until assayed.

### Antibody analysis

Determination of antibodies to CPV-2 in serum samples was carried out with a commercial kit (Parvo Ab ELISA, AGROLABO, Scarmagno, TO, Italy) consisting of an indirect immunoenzymatic assay with spectrophotometric reading (450 nm), which had been validated by the manufacturer in relation to the ‘gold standard’ haemagglutination inhibition test with a declared sensitivity of 95% and specificity of 98.5%. The sample optical density/positive control optical density (S/P) ratio was calculated. According to the suggested cut-off, sera with S/*P* values lower than 0.15 were detected as CPV-2 negative, while samples with S/P values higher than 0.15 were classified as CPV-2 positive. The S/P value was used, according to the manufacturer’s instructions, for the calculation of antibody titres using the following formula: Antibody titres = 54(e^^4(S/P)^). A value of 1:100 was considered the minimum protective titre by the manufacturer.

### Statistical analysis

All data were analysed using GraphPad Prism (vers. 6; GraphPad Software, California, USA). The normality of distribution was tested using Kolmogorov and Smirnov tests.

The number of months that had elapsed since the last vaccination was calculated for each animal.

The correlation between months from vaccination and antibody titre was calculated by means of Spearman’s test. An arbitrary value of 25,000 was attributed to out-of-scale antibody titres.

The time elapsed since the last vaccination was categorized in the following classes: < 12 months; 13–24 months; 25–36 months; 37–48 months; and > 49 months. Antibody titres for each class were compared using the Kruskal-Wallis test, followed by Dunn’s post-tests.

The Mann-Whitney test was applied to compare the time from vaccination in animals showing values higher or lower than the cut-off antibody titre and between males and females.

Values of *P* < 0.05 were considered statistically significant.

## Data Availability

The datasets used and/or analysed during the current study are available from the corresponding author on reasonable request.

## Abbreviations

CPV-2: Canine parvovirus type 2
FPV: Feline parvovirus
MLV: Modified live virus
